# Profiling of Human Molecular Pathways Affected by Retrotransposons at the Level of Regulation by Transcription Factor Proteins

**DOI:** 10.3389/fimmu.2018.00030

**Published:** 2018-01-30

**Authors:** Daniil Nikitin, Dmitry Penzar, Andrew Garazha, Maxim Sorokin, Victor Tkachev, Nicolas Borisov, Alexander Poltorak, Vladimir Prassolov, Anton A. Buzdin

**Affiliations:** ^1^Engelhardt Institute of Molecular Biology, Russian Academy of Sciences, Moscow, Russia; ^2^D. Rogachev Federal Research Center of Pediatric Hematology, Oncology and Immunology, Moscow, Russia; ^3^The Faculty of Bioengineering and Bioinformatics, Lomonosov Moscow State University, Moscow, Russia; ^4^OmicsWay Corp., Walnut, CA, United States; ^5^National Research Centre Kurchatov Institute, Centre for Convergence of Nano-, Bio-, Information and Cognitive Sciences and Technologies, Moscow, Russia; ^6^Shemyakin-Ovchinnikov Institute of Bioorganic Chemistry, Moscow, Russia; ^7^Program in Immunology, Sackler Graduate School, Tufts University, Boston, MA, United States

**Keywords:** endogenous retrovirus, transcription factor binding site, retrotransposon, retroelement, molecular pathway, immunity, evolution, human genome evolution

## Abstract

Endogenous retroviruses and retrotransposons also termed retroelements (REs) are mobile genetic elements that were active until recently in human genome evolution. REs regulate gene expression by actively reshaping chromatin structure or by directly providing transcription factor binding sites (TFBSs). We aimed to identify molecular processes most deeply impacted by the REs in human cells at the level of TFBS regulation. By using ENCODE data, we identified ~2 million TFBS overlapping with putatively regulation-competent human REs located in 5-kb gene promoter neighborhood (~17% of all TFBS in promoter neighborhoods; ~9% of all RE-linked TFBS). Most of REs hosting TFBS were highly diverged repeats, and for the evolutionary young (0–8% diverged) elements we identified only ~7% of all RE-linked TFBS. The gene-specific distributions of RE-linked TFBS generally correlated with the distributions for all TFBS. However, several groups of molecular processes were highly enriched in the RE-linked TFBS regulation. They were strongly connected with the immunity and response to pathogens, with the negative regulation of gene transcription, ubiquitination, and protein degradation, extracellular matrix organization, regulation of STAT signaling, fatty acids metabolism, regulation of GTPase activity, protein targeting to Golgi, regulation of cell division and differentiation, development and functioning of perception organs and reproductive system. By contrast, the processes most weakly affected by the REs were linked with the conservative aspects of embryo development. We also identified differences in the regulation features by the younger and older fractions of the REs. The regulation by the older fraction of the REs was linked mainly with the immunity, cell adhesion, cAMP, IGF1R, Notch, Wnt, and integrin signaling, neuronal development, chondroitin sulfate and heparin metabolism, and endocytosis. The younger REs regulate other aspects of immunity, cell cycle progression and apoptosis, PDGF, TGF beta, EGFR, and p38 signaling, transcriptional repression, structure of nuclear lumen, catabolism of phospholipids, and heterocyclic molecules, insulin and AMPK signaling, retrograde Golgi-ER transport, and estrogen signaling. The immunity-linked pathways were highly represented in both categories, but their functional roles were different and did not overlap. Our results point to the most quickly evolving molecular pathways in the recent and ancient evolution of human genome.

## Introduction

Retrotransposable elements (REs) are mobile genetic elements that self-reproduce in the host DNA. For proliferation of their copies, they use a specific molecular mechanism based on RNA-dependent synthesis of DNA by an enzyme termed reverse transcriptase (RT) (1). Taken together, REs occupy ~40% of human DNA. They are represented by three major classes: human endogenous retroviruses/LTR reprotransposons (HERV/LRs) and LINE and SINE retrotransposons (2). The first group shaped ~8% of human genome, whereas LINEs and SINEs ~20 and 13%, respectively. HERV/LRs are thought to be remnants of multiple previous retroviral infections (3, 4). Unlike many common infectious retroviruses, they became inheritable because their insertions occurred in the ancestral germ cells (5). By contrast, LINEs and SINEs are non-infective retrotransposons. HERV/LRs and LINEs are called autonomous mobile elements because they encode RT, and SINEs—non-autonomous because for their life cycle they use foreign, LINE-encoded enzymes (6).

The studies of evolutionary dynamics of REs revealed that they were actively proliferating in human DNA until the most recent events in human speciation (7). All groups of REs include transcription of their genomic copies as the necessary step in their life cycle. Therefore, RE sequences are enriched in transcription factor binding sites (TFBSs) and other regulatory motifs (8–11). Moreover, most of the RE copies accumulated mutations and could strengthen their regulatory repertoire. For example, the HERV/LRs include promoters (12), enhancers (13, 14), polyadenylation signals (5), chromatin folding reshapers (15), and binding sites for various nuclear proteins (16). In human genome, REs are represented by millions of individual elements that can be found in the vicinity of any known gene. Therefore, the REs are considered among the major factors of evolution of gene expression regulatory networks. For example, ~30% of all transcriptional factor p53 binding sites in the human genome fall within the HERV/LR elements (17). We recently showed that functional TFBS within the human-specific endogenous retroviruses may control expression of schizophrenia-linked gene PRODH in human hippocampus (14).

Transcription factor binding sites denote regulatory fragments of DNA that can bind transcription factors and influence gene expression. Congruently, mapping DNaseI hypersensitivity sites (DHS) became a golden standard for the identification of regulatory loci of an open chromatin (18). Recent studies evidence that huge numbers of DHS and TFBS in the human genome are located within the TEs. For example, totally, ~155,000 and ~320,000 HERV/LR-derived DHS and TFBS were identified, respectively (19). For the HERV/LR elements, ~110,000 inserts (~15%) had at least 2 TFBS and ~140,000 individual inserts (~19%)—at least 1 DHS, as shown in the previous report (19). Finally, at least ~31% of all mapped human transcription start sites were identified within the REs (20).

Like never before, high-throughput mapping of functional genomic features such as TFBS, DHS, and different types of histone binding sites provides opportunity to explore RE influence on gene expression in a comprehensive way. Besides individual affected genes, their functional groups can be assayed, including gene families and molecular pathways. Intracellular molecular pathways are involved in all major events in the living organisms. The major groups are metabolic, cell signaling, cytoskeleton reorganization, and DNA repair pathways (21, 22).

The pathways may include tens or hundreds of nodes and aggregate up to several hundreds of different gene products (23, 24). Remarkably, each node in a pathway is typically built not by just a single-gene product, but rather by their groups. Those can be formed by the homologous families of similarly functionally charged proteins, or by the various protein subunits which may be all needed to execute a function required for the pathway activity (25, 26).

For few decades, the molecular pathways are still on the forefront of biomedical sciences (27–30). Hundreds of thousands of molecular interactions and thousands of molecular pathways have been discovered by the molecular biologists and cataloged in different databases (31–37).

On the other hand, the gene products can be sorted according to their functional role in the cell and with reference to the molecular or supramolecular processes they are involved. This way of data aggregation does not require knowledge of the particular chains of molecular interactions, as for the above group of the pathway databases. For example, the gene ontology (GO) database provides functional and structural labels to the gene products or their groups.^1^ By uploading a specific set of gene products, one can find it out whether this list is statistically significantly enriched in certain types of functional gene families. For example, in certain applications this enables to make a quick overview of the differentially expressed and most frequently mutated groups of genes (38).

In this study, we aimed to identify molecular processes most deeply regulated by the RE inserts in the human cells. To this end, we mapped the available TFBS data on the individual human REs for K562 cells. We found that in the close gene neighborhood, ~17% of TFBS overlap with the RE sequences, of them 44% belong SINEs, 33%—to LINEs, and 23%—to LR/ERVs. Most of the REs hosting TFBS were highly diverged repeats, and for the evolutionary young (0–8% diverged) elements we identified only ~7% of all RE-specific TFBS. Among them, SINEs hosted ~68%, LINEs ~15%, and LR/ERVs ~17% of TFBS.

Depending on the number of RE-mapped TFBS in the vicinities of the particular genes, we calculated a score for each gene positively reflecting the RE impact on gene regulation. Based on the scores for the individual genes, for the first time we could identify the molecular processes most strongly impacted by the RE regulatory features. To this end, we applied and modified bioinformatic method Oncofinder that has been used before only for the analysis of gene or microRNA expression profiles (39) and could effectively reduce experimental noise caused by different experimental platforms and batch effects (40, 41). In the initial version, this method makes it possible to calculate the quantitative value reflecting molecular pathway activation, called pathway activation strength (PAS). The absolute value of PAS reflects the extent of a molecular pathway perturbation. Negative PAS values indicate downregulation of molecular pathways, positive values mean upregulation, whereas 0 values represent non-significant difference with the control samples (42). Previously PAS values were calculated only based on the gene expression profiles (high-throughput mRNA or protein levels). Here, we for the first time applied this rationale to quantitatively measure the impact of REs on the evolution of human molecular pathways with the input data on TFBS distribution.

We found that the gene-specific distributions of the RE-linked TFBS generally correlated with the distributions for all the TFBS. However, several groups of molecular processes were highly enriched in the RE-linked TFBS regulation. They were strongly connected with the immunity and response to pathogens, with the negative regulation of gene transcription, ubiquitination, and protein degradation, extracellular matrix organization, regulation of STAT signaling, fatty acids metabolism, regulation of GTPase activity, protein targeting to Golgi, regulation of cell division and differentiation, and with development and functioning of perception organs and the reproductive system. By contrast, the processes most weakly implicated by the REs were linked mainly with the embryonic development.

We also found that both the gene- and pathway-specific scores significantly correlated for the evolutionary *young* and *all* RE-linked TFBS, thus evidencing that the major evolutional trends in RE-linked TFBS regulation are largely conserved in the evolution. However, we identified many differences in the regulation features by the younger and older fractions of the REs. The regulation by the older fraction of the REs was linked mainly with the immunity, cell adhesion, Notch, Wnt, and integrin signaling, neuronal development and sensing, chondroitin sulfate and heparin metabolism, cAMP metabolism, endocytosis, and IGF1R signaling.

By contrast, the younger REs were regulating the other aspects of immunity, cell cycle progression and apoptosis attenuation, PDGF, TGF beta, EGFR, and p38 signaling, histone deacetylation and DNA methylation interplay, structure of nuclear lumen, metabolism (primarily catabolism) of phospholipids and heterocyclic nitrogen-containing molecules, insulin and AMPK signaling, retrograde Golgi-ER transport, estrogen signaling, and oocyte maturation. The immunity-linked pathways were highly represented in both categories (recently and long-term evolving), but their functional characteristics were different and did not overlap. Our results shed light on the evolution of regulatory network in humans and point to the most quickly evolving molecular pathways in higher primates.

## Results

### Mapping of RE-Specific Human TFBS

**Figure 1:**
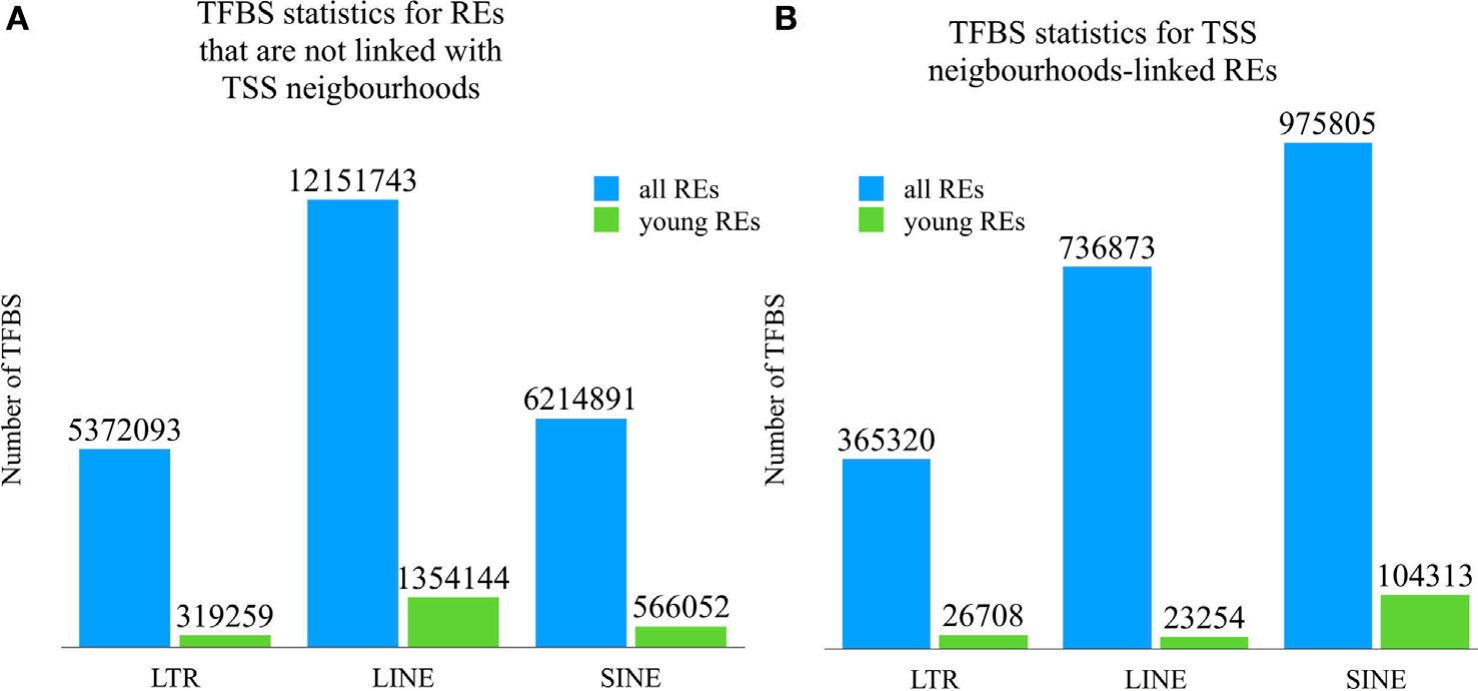
Distribution of RE-linked transcription factor binding site (TFBS) **(A)** outside and **(B)** inside 10 kb neighborhoods of TSS between the different groups of REs. Numbers are given for the mapped TFBS of each category. Green columns denote TFBS for the evolutionary young REs (0–8% divergence from the respective consensus sequence). Blue columns show TFBS distribution for the fraction of all REs.

From the ENCODE database, we extracted TFBS information for the human myelogenous leukemia cell line K562. The TFBS data for different transcription factor proteins were based on the sequencing of immunoprecipitated DNA fragments (43, 44). The cell line K562 was chosen because it was assayed for the maximum number of transcription factor proteins (225 versus only 120 in the next by abundance cell line GM12878). The TFBSs for all available transcription factor proteins were then mapped onto genomic sequences of the human REs. To identify a fraction of TFBS most likely involved in the regulation of gene expression, we took the elements located in the 5-kb neighborhood of the transcription start sites of known protein-coding genes. A total of 13,029,963 TFBS reads were identified close to transcription start sites. Among them, 2,232,273 (~17%) overlapped with the RE sequences and were referred as the RE-specific fraction of TFBS. Among them, ~44% were attributed to SINEs; ~33%—to LINEs, and 23%—to LR/ERVs. Most of the REs hosting TFBS were highly diverged repeats. For the evolutionary younger REs (0–8% diverged from their consensus sequence), we identified 154,275 TFBS (~7% of all RE-specific TFBS). Among them, SINEs hosted ~68%, LINEs ~15%, and LR/ERVs ~17% of the RE-specific TFBS (Figure 1B). The analogous distribution of RE-linked TFBS outside the gene promoter neighborhoods (the rest of the genome) is shown on Figure 1A. Interestingly, our data strongly suggest that there is a bias against active TFBS within the evolutionary young LINE elements located close to the gene promoters (Figure 1B).

For the same 5-kb neighborhood, we next calculated relative concentration of RE-linked TFBS per kilobase for different RE classes (Table 1). In may be seen that for the *young* elements, their ability to provide functional TFBS is generally ~14 times lower than for the group of *all* REs. For the LR/ERVs this factor is also ~14-fold lower, whereas for the LINEs and SINEs ~32- and 9-fold lower, respectively. The extent of this suppression was different for the different types of REs varying from ~9-fold for SINEs till ~32-fold for LINEs, with the median level for LR/ERVs (Table 1). The absolute concentrations for the REs were also different, varying from ~0.1 for LR/ERVs and LINEs till 0.4 for SINEs.

**Table 1:** Relative concentration of RE-linked transcription factor binding site (TFBS) in 5-kb neighborhood of human gene transcription start sites.

Concentration of TFBS per kilobase			

Class of RE	All	Young	Fold change
LINE	2.939	0.093	−31.6
SINE	3.892	0.416	−9.4
LR/ERV	1.457	0.107	−13.6
Total REs	8.287	0.616	−13.5

### Identification of Human Genes Impacted by RE-Linked TFBS

**Figure 2:**
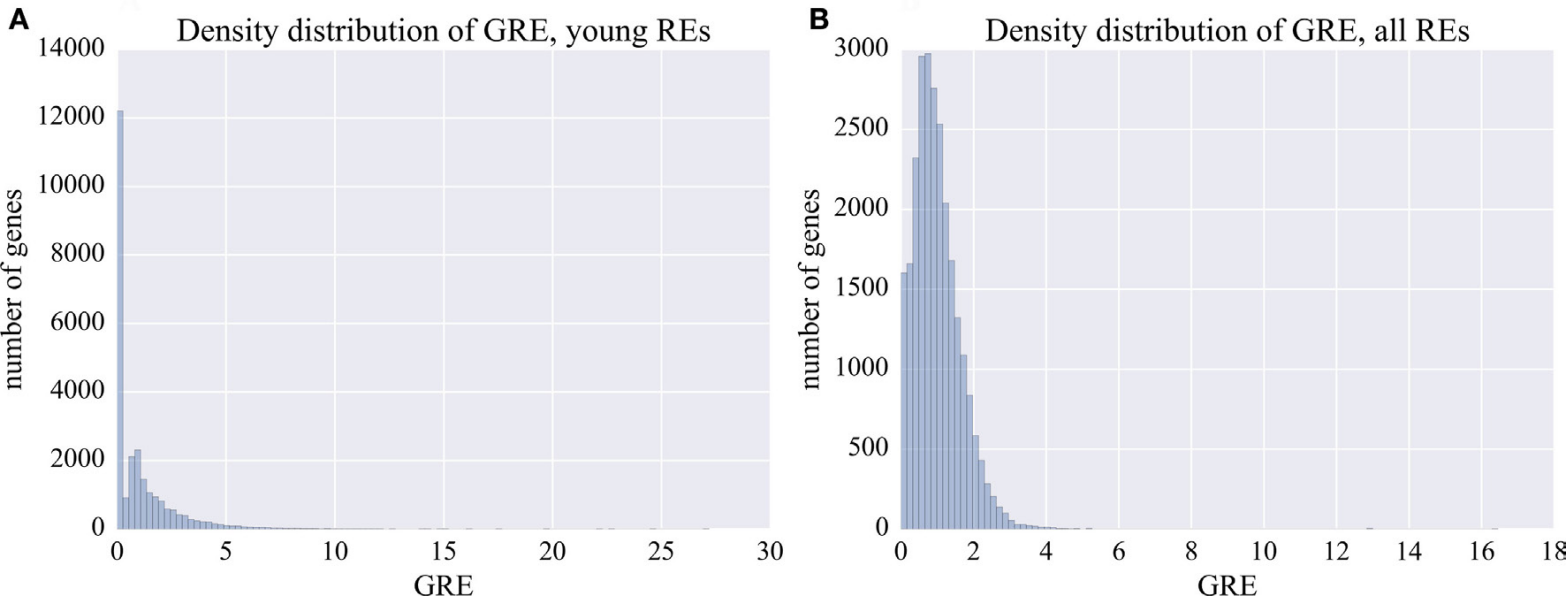
Distribution of GRE score among the known human genes. **(A)** Distribution of GRE for the young fraction of REs (0–8% divergence from the respective consensus sequence). **(B)** Distribution of GRE for the total fraction of all REs.

For every individual gene, we calculated its enrichment score for the RE-linked TFBS. We introduced the value termed *Gene RT-linked TFBS enrichment score (TES)* or *GRE score* (Figure 2). GRE is the sum of RE-specific TFBS reads mapped close to the individual gene’s transcriptional start site, normalized on the average sum of RE-specific TFBS reads for all genes. For every individual gene, GRE score is calculated according to the formula:

$${\text{GRE}}_{g}=\frac{{\text{TES}}_{g}}{\frac{1}{n}\sum_{i=1}^{n}{\text{TES}}_{i}},$$

where GRE*_g_* is the GRE score for a gene *g*; TES*_g_* is the number of RE-linked TFBS reads for a gene *g*; *i* is gene index and TES*_i_* is the number of RE-linked TFBS reads for a gene *i*; and *n* is the total number of genes.

For every gene, the GRE score makes it possible to measure the extent of enrichment by the RE-linked regulatory elements. For example, GRE = 1 means average impact on the regulation of a gene. GRE > 1 means that the individual gene is enriched in RE-specific TFBS. Contrarily, GRE < 1 means that the gene has lower than average number of RE-specific TFBS.

Our results suggest that there is a fraction of human genes highly enriched in the content of RE-specific TFBS in the regulatory regions, which is reflected by high GRE scores of up to 5 for the protein-coding genes (Table S1 in Supplementary Material; Figure 2). By contrast, many other genes had close to 0 GRE values (Figure 2).

While GRE provides an integral assessment of TFBS impact belonging to all 225 TFs studied here, we also elucidated how strongly each specific TF affects expression of each specific human gene *via* gene-linked REs. For each gene, *i* and TF *j* an entry with indices (*i, j*) is number of RE-linked TFBS of this TF in the neighborhood of this gene. Our results suggest that most human genes are affected by RE-linked TFBS of various different TFs (Table S2 in Supplementary Material).

### Identification of Molecular Pathways Impacted by RE-Linked TFBS

To assess the impact of RE-linked TFBS on the regulation of molecular pathways, we introduced a quantitative metric termed *Pathway Involvement Index* (*PII*) that is calculated according to the following formula:

$${\text{PII}}_{p}=\frac{\sum_{i=1}^{n}{\text{GRE}}_{i}}{n},$$

where PII*_p_* is the PII score for a pathway *p*; GRE*_i_* is the GRE score for a gene *I*; and *n* is the number of genes in a pathway *p*. To avoid misleading higher PII values for bigger pathways, PII*_p_* value is normalized on the number of genes in a pathway.

For information about gene products forming molecular pathways, we used the databases BioCarta, KEGG, NCI, Reactome, and Pathway Central. For our profiling, we used 1,749 molecular pathways covering ~11,000 human genes.

The biggest PII scores suggested the highest impact of RE-linked TFBS on the regulation of the whole molecular pathway, and *vice versa*. Zero PII score means no impact on the regulation of the molecular pathway. Similarly to the figure observed for the individual genes, the distribution of PII scores suggests that many molecular pathways are enriched in the regulatory motifs contributed by the REs. We next attempted to characterize the most strongly impacted individual genes and molecular pathways.

### Genes Impacted by the RE-Linked TFBS Regulation

The human genes were sorted according to their GRE scores. For different genes, they varied from 0 to 16.4 (Figure 2B; Table S1 in Supplementary Material). The top and the bottom 6% of the genes with the highest and the lowest GRE scores profiled for all REs were next analyzed using GO annotation terms and DAVID software.

#### Top Genes

For the top 6% genes, we identified 48 significantly enriched annotation clusters (Table S3 in Supplementary Material). Among them, 8 (17%) were connected with ribosome biogenesis and translation, 7 (15%) with protein complex assemblies, 5 (10%) with chromatin organization and maintaining structure of the nucleus, 5 (10%) with cell stress and innate immune response mechanisms, 3 (6%) with microtubules and organization of mitotic spindle, 3 (6%) with the regulation of programmed cell death, 3 (6%) with oxidoreductase activity involving purine nucleosides, 2 (4%) with DNA replication and repair, 2 (4%) with formation of mitochondrial outer membrane complexes, and 2 (4%) with the regulation of autophagy. One cluster represented p53-regulated signal transduction, another one—maintaining nucleolus structure. Other features were also presented by minor number of clusters.

#### Bottom Genes

For the least RE-impacted genes with close to zero RE scores (bottom 6%), quite distinct set of 96 annotation clusters was observed (Table S3 in Supplementary Material). Among them, notably high proportion was taken by 80 (83%) clusters directly linked with embryonic development. Among the others, 8% represented different transcription factor binding assemblies, 2% neuronal axon development, 2% cell–cell adhesion, and signaling, and 2% positive regulation of cell proliferation.

### Molecular Pathways Impacted by the RE-Linked TFBS Regulation

We next ranked the molecular pathways by their enrichment with the RE-linked TFBS. For the analysis, we took the molecular pathways each including at least 10 gene products. The pathways were ranked according to their PII scores (Table S4 in Supplementary Material). We analyzed 65 top (pathways with the highest PII score) and 65 bottom (pathways with the lowest PII score) molecular pathways.

#### Top Pathways

The following groups of top molecular pathways were identified: 15 (24%) pathways linked with DNA replication and repair, 19% for ribosome and translation, 11% for cytoskeleton remodeling and cell migration, 10% for nuclear transport of mRNA, 10% for other types of nuclear trafficking, 6% for cell stress and innate immune response, 6% for cellular export machinery and vesicle trafficking, 3% for regulation of microtubules and mitotic spindle assembly, 3% for mRNA decay mechanisms, and 8% for the other processes (Table S5 in Supplementary Material).

The major featured molecular processes dealt with protein translation, cell stress and innate immune response, cytoskeleton remodeling, and DNA replication and repair.

#### Bottom Pathways

The following groups of molecular pathways had the lowest PII scores (Table S5 in Supplementary Material): 18 (30%) for extracellular matrix and cell migration, 16% for interleukin-related cell signaling, 21% for neurogenesis, 15% for embryogenesis and morphogenesis, 3% for PTEN signaling, 3% related to G protein coupled receptors (GPCR) signaling, 3% for fatty acids metabolism, and 9% for the other processes.

### Comparison of RE-Linked and Non-RE-Linked TFBS Profiles

However, it appeared unclear whether the genes/pathways were enriched in RE-linked TFBS congruently with the overall (not RE-specific) TFBS distribution. To characterize total TFBS distribution trends, we introduced a relative value termed GTE (Gene TFBS Enrichment). GTE is expressed by the following formula:

$${\text{GTE}}_{g}=\frac{{\text{TTS}}_{g}}{{\text{TTS}}_{m}},$$

where TTS*_g_* is total number of TFBS reads mapped in the 5-kb neighborhood of a gene *g* and TTS*_m_* is the mean TTS for all genes under investigation. To define RE-specific enrichment in the regulation of an individual gene, a relative value termed NGRE was introduced:

$${\text{NGRE}}_{g}{=\text{GRE}}_{g}{/\text{GTE}}_{g}.$$

Bigger NGRE value means bigger impact of RE-specific regulation of certain gene, and *vice versa*.

Another set of values was introduced to estimate the relative RE-specific impact in the regulation of molecular pathways. We added a metric termed PGI (Pathway Gene-based TFBS Index) to assess the impact of total TFBS on the regulation of molecular pathways:

$${\text{PGI}}_{p}=\frac{\sum_{i=1}^{n}{\text{GTE}}_{i}}{n},$$

where PGI*_p_* is the PGI score for a pathway *p*; GTE*_i_* is the GTE score for a gene *I*; and *n* is the number of genes in a pathway *p*.

In turn, the normalized PII called NPII determines enrichment in RE-specific TFBS regulation of a molecular pathway:

$${\text{NPII}}_{p}={\text{PII}}_{p}{/\text{PGI}}_{p}.$$

where PII*_p_* is a Pathway RE-based Involvement Index for a pathway *p* and PGI*_p_* is the Pathway Gene-based TFBS Index for a pathway *p*.

**Figure 3:**
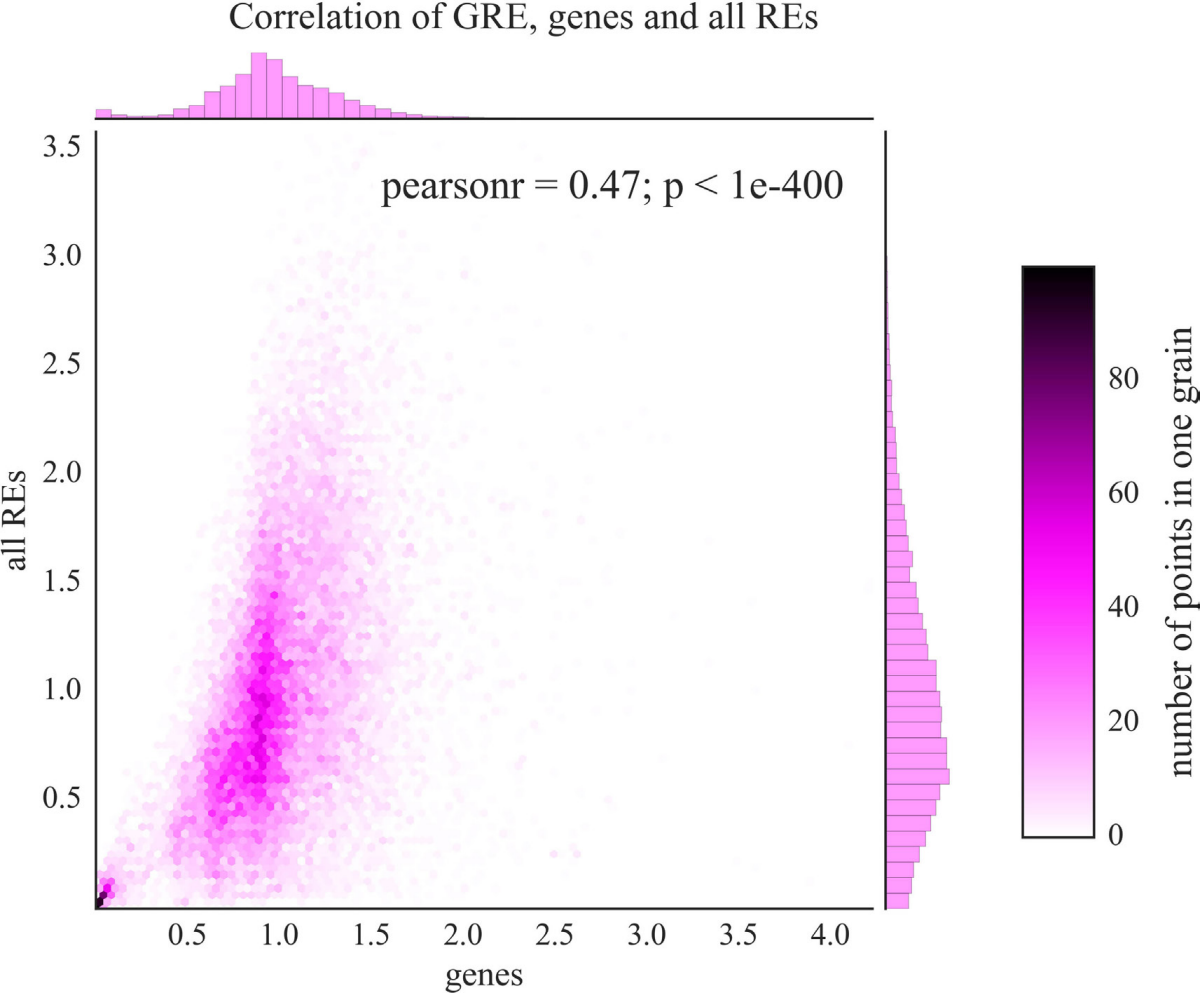
Comparison of GRE scores (axis of ordinates) and GTE scores (abscissa axis) for known human genes. Color scale is given to show densities of incidences on the plot. Each dot represents a single gene. Pearson *r*—Pearson correlation coefficient; *p*—Pearson *p*-value.

At the level of individual genes, we observed statistically significant correlations between the GRE (based on RE-linked TFBS) and GTE (based on all TFBS) scores (Figure 3, Pearson correlation coefficient = 0.47, *p*-value < 0.001; Table S6 in Supplementary Material). The respective lists of top and bottom GO annotation terms were also highly interconnected featuring protein translation, chromatin remodeling and DNA replication as the most strongly regulated processes, whereas neurogenesis, GPCR signaling, and developmental programs were the most weakly regulated aspects. Taken together, these data evidence that the abundance of RE-linked TFBS roughly (correlation = 0.47) follows overall trend of all TFBS accumulation near gene promoter regions.

### Genes and Pathways under Strong Regulation by the REs

To assess the specific trends in RE-dependent regulation of gene expression, we analyzed distributions of the NGRE scores, which characterize the impact of RE-specific TFBS normalized on the regulation by all TFBS for the individual genes (Table S7 in Supplementary Material). The most strongly specifically regulated protein-coding genes were *USP176L26, USP17L13*, and *USP17L12* for ubiquitin-specific peptidases. We next analyzed the lists of 6% top and bottom genes sorted according to NGRE (Table 2). The top GO features were linked with immunity and response to pathogens (64/295 terms, or 32%), 7% for organ development, 6% for negative regulation of gene transcription, 6% for chromatin assembly, 6% for protein targeting to Golgi, 4% for ubiquitination and protein degradation, 4% for extracellular matrix organization, 4% for regulation of STAT signaling, 4% for perception organ development and functioning, 4% for negative regulation of macromolecule metabolism, 3% for peptide modifications, 3% for regulation of GTPase activity, 3% for reproductive systems development and functioning, 3% for negative regulation of cell differentiation and positive regulation of cell division, 2% for regulation of body fluids, and 9% was for the other processes (Table S8 in Supplementary Material).

**Table 2:** Gene ontology (GO) functional annotation clusters in top and bottom 6% of human genes sorted by their NGRE scores.

Cluster, GO terms	Percentage of clusters	
	Top 6%	Bottom 6%
Immunity and response to pathogens	32	–
Organ development and embryogenesis	7	45
Gene transcription and negative regulation	6	–
Chromatin assembly	6	16
Protein targeting to Golgi	6	–
Ubiquitination and protein degradation	4	–
Extracellular matrix organization	4	–
Regulation of STAT signaling	4	–
Perception organ development	4	–
Negative regulation of metabolism	4	–
Peptide modifications	3	–
Regulation of GTPases	3	–
Reproductive system development	3	–
Regulation of differentiation and cell proliferation	3	2
Regulation of body fluids	2	–
Transcription and processing of RNA	–	16
Ribosome assembly and protein translation regulation	–	8
Regulation of apoptosis	–	2
Proteasomal degradation	–	2
Steroid receptor signaling	–	2

For the group of the bottom 6% of genes, the least regulated features were linked to embryonic development and stem cell differentiation (44/98, or 45%), 16% for transcription and processing of RNA, 16% for nuclear chromatin organization, 8% for ribosome functioning and protein translation, 2% for regulation of apoptosis, 2% for ubiquitin binding, 2% for steroid receptor signaling, 2% for regulation of cell proliferation, and 7% for the other activities (Table 3; Table S8 in Supplementary Material).

**Table 3:** Functional groups of top and bottom molecular pathways sorted by their NPII scores.

Functional group	Percentage	
	Top 6%	Bottom 6%
Fatty acids metabolism	19	–
Immunity and response to pathogens	15	14
Nuclear transport	9	–
Maturation of RNA (mRNA and small RNAs)	9	–
DNA repair and replication	6	–
Alpha-synuclein signaling	5	–
Ubiquitination and protein degradation	3	–
Protein targeting to Golgi	3	–
Nerve growth and neuronal signaling	–	24
Organ development, embryogenesis, and cell adhesion	–	35
IGF1R signaling and regulation of glucose metabolism	–	9

Similar tendencies were seen at the level of molecular pathways (Table 3; Table S9 in Supplementary Material). NPII scores were calculated that reflect the RE-specific impact on the regulation of molecular pathways normalized to the impact by all TFBS. The top 65 pathways sorted according to NPII score were linked with fatty acids metabolism (19%), immunity and pathogen recognition (15%), nuclear transport (9%), maturation of mRNA (6%), DNA repair and replication (6%), synuclein A signaling (5%), small RNA biogenesis and function (3%), protein ubiquitination (3%) protein trafficking to Golgi (3%), and other pathways.

The major bottom pathways were involved in the regulation of nerve growth and neuronal signaling (24%), cell adhesion (19%), cytokine networks (14%), other developmental programs (14%), IGF signaling, and regulation of glucose metabolism (9%) (Table 3; Table S9 in Supplementary Material).

We next compared the NGRE score distribution at the gene level and NPII score distribution at the pathway level for the fractions of *all* REs and evolutionary younger REs (*young*; 0–8% diverged from their consensus sequence).

**Figure 4:**
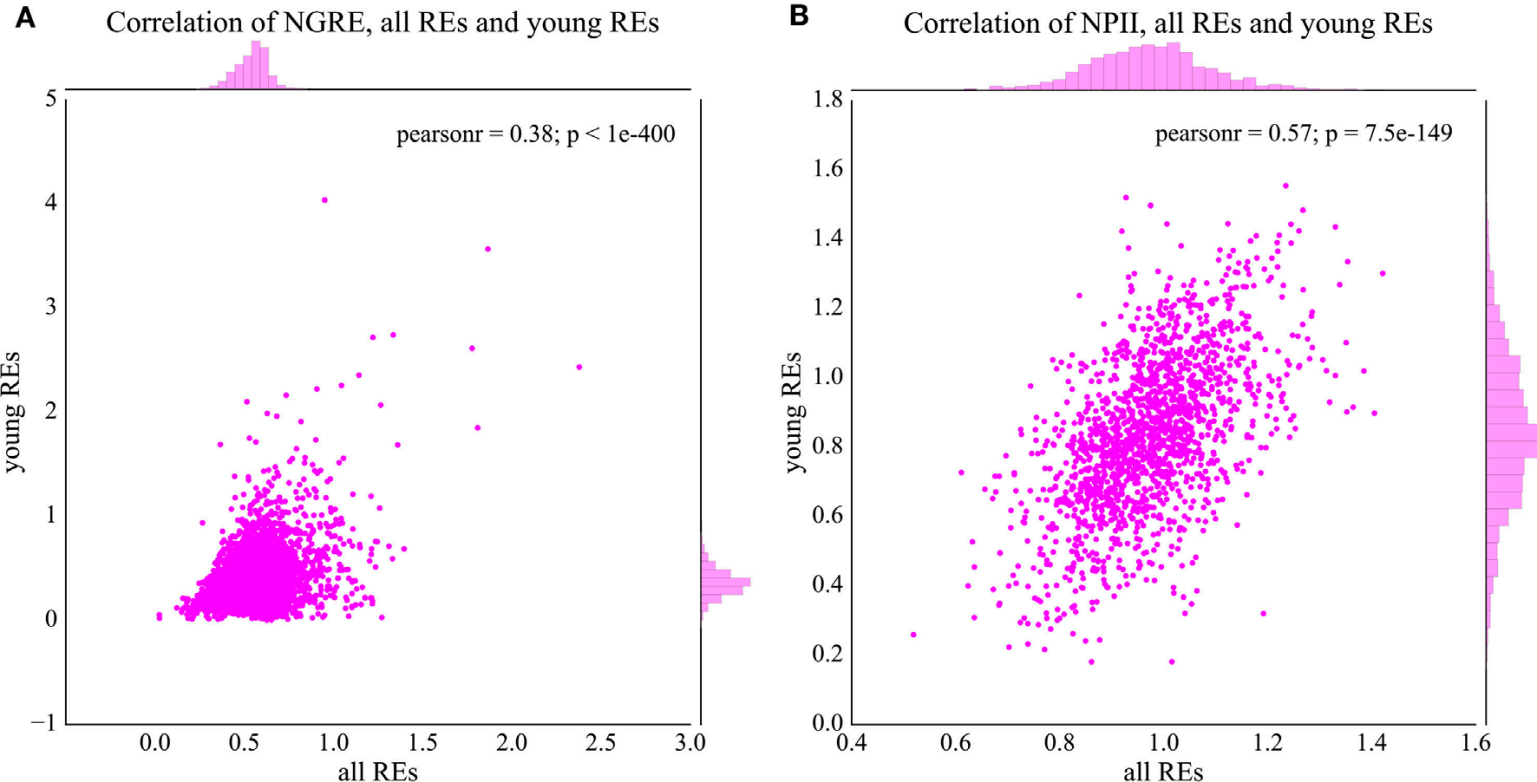
Comparison of normalized transcription factor binding site distributions between the young (0–8% divergence from the respective consensus sequence) and total fractions of REs. **(A)** Comparison of NGRE scores (gene level of regulation), each dot represents a single gene. **(B)** Comparison of NPII scores (pathway level of regulation), each dot represents a single molecular pathway. Pearson *r*—Pearson correlation coefficient; *p*—Pearson *p*-value.

In general, NPII and NGRE scores were statistically significantly correlated for the *young* and *all* REs, but the pathway-linked NPII scores showed bigger correlation (Figure 4A, Pearson correlation coefficient = 0.38, *p*-value < 0.001; Figure 4B, Pearson correlation coefficient = 0.57, *p*-value < 0.001). These data are congruent with the previous findings that the data aggregation at the level of molecular pathways frequently provides more congruent results compared with the single-gene level of analysis (41), especially in the case of cancer (45) and neurodegenerative diseases (46, 47).

Although there was a 0.38–0.57 correlation (Figure 4), some regulatory features were different between the *young* and *all* REs. To analyze the differences in pathway regulation by *all* and *young* REs, we calculated *ratio* of *all* and *young* REs separately for the NGRE and the NPII scores. Bigger values here mean greater regulation changes in a long-term rather than recent evolution; lower values mean greater changes in the recent evolution (Table 4; Tables S10 and S11 in Supplementary Material for all/young ratio of NGRE and NPII, respectively). In the long-term (but not short-term) perspective, the *top* 65 pathways sorted according to NPII ratio were dealing mainly with cell adhesion, Notch, Wnt, and integrin signaling (20%), immunity and cytokine signaling (20%), neuronal development and sensing (17%), chondroitin sulfate and heparin metabolism (8%), cAMP metabolism (6%), endocytosis (3%), and IGF1R signaling (3%).

**Table 4:** Functional groups of top and bottom molecular pathways sorted by the ratios of NPII scores for the *all* and *young* RE-linked transcription factor binding sites.

Functional group	Percentage	
	Top 6%	Bottom 6%
Cell adhesion, Notch, Wnt, and integrin signaling	20	–
Immunity and response to pathogens	20	17
Nerve growth and neuronal signaling	17	–
Metabolism of chondroitin sulfate and heparin	8	–
Metabolism of cAMP	6	–
Endocytosis	3	–
IGF1R signaling and regulation of glucose metabolism	3	–
Cell cycle progression and regulation of apoptosis	–	21
PDGF, TGF beta, EGFR, and p38 signaling	–	12
Histone deacetylation and DNA methylation	–	10
Phospholipid metabolism	–	9
Insulin and AMPK signaling	–	6
Protein targeting to Golgi	–	3
Estrogen signaling and oocyte maturation	–	3

The *lower scoring* pathways (most quickly evolving in the recent evolution) were linked mainly with the general cell cycle progression and apoptosis attenuation mechanisms (21%), immunity (17%), PDGF, TGF beta, EGFR, and p38 signaling (12%), histone deacetylation and DNA methylation interplay (10%), phospholipid metabolism (9%), insulin and AMPK signaling (6%), retrograde Golgi-ER transport (3%), and estrogen signaling and oocyte maturation (3%).

Sorting according to NGRE ratio had no sense for the *top* individual genes because there were too many 0 values on the denominator for the NGRE scores calculated for the *young* REs. However, the list of the *bottom* 6% of genes was successfully generated presumably including the most quickly evolving genes in the recent human evolution (according to RE-linked TFBS acquisition). These genes were mostly involved in the catabolism and synthesis of heterocyclic nitrogen-containing molecules and phospholipids metabolism (50/163, or 31%), nuclear lumen structure (8%), mRNA splicing and processing (7%), ribosome assembly and translation (7%), DNA and histone methylation (4%), and DNA repair (2%).

## Materials and Methods

### Identification of RE-Specific TFBSs

Complete genome binding profiles of 225 transcription factor proteins were extracted from the ENCODE database^2^ for human cell line K562 according to the standard ENCODE ChIP-seq protocol (43). The reference human genome assembly 2009 (hg19) was indexed using Burrows–Wheeler algorithm using BWA software.^3^ Concatenation of fastq files with single-end or pairwise reads, alignment to the reference genome, and filtering were done using BWA, Samtools, Picard, Bedtools, and Phantompeakqualtools software.^4^ The aligned TFBS were mapped on the RE sequences annotated by RepeatMasker^5^ and downloaded from the USCS Browser^6^ (RepeatMasker table). TFBS occurrence data were extracted from the bedGraph files.^7^ The fold change over control profiles for TFBS, as well as the profiles for *p*-value to reject the null hypothesis that the signal at that location, is present in the control were built using Macs software^8^ based on the alignment data. The list of transcription factors investigated and the raw data obtained from the ENCODE web site are shown on the Tables S12 and S13 in Supplementary Material.

For every individual mapped RE, we calculated the TES according to the formula:

$$\text{TES}=\sum_{i=1}^{225}b_{i},$$

where *b_i_* is the number of TFBS reads for transcription factor *i* mapped on the individual RE.

### Measuring Gene Enrichment by the RE-Linked TFBS

The coordinates of human protein-coding genes were downloaded from the USCS Browser.^9^ For each gene, all individual REs overlapping with the 5-kb long neighborhood of its reference transcription start site were selected for further analysis. The 5-kb neighborhood covered an interval starting 5 kb upstream and ending 5 kb downstream the transcription start site. The selected REs were classified according to their structure (HERV/LR, LINE, and SINE) and divergence from the consensus sequence for the respective RE family. The REs with the divergence less that 8% were considered “young” elements. We introduced an integral enrichment score to calculate the RE-linked TFBS enrichment specific to every individual gene (GRE score):

$${\text{GRE}}_{g}=\frac{{\text{TES}}_{g}}{\frac{1}{n}\sum_{i=1}^{n}{\text{TES}}_{i}},$$

where GRE*_g_* is the GRE score for a gene *g*; TES*_g_* is the sum of TES values for all the RE types for the REs located in the 5-kb neighborhood of a gene *g*; *n* is the number of all genes; and *i* is gene index and TES*_i_* is the sum of TES values for all the RE types for the REs located in the 5-kb neighborhood of a gene *i*. Alternatively, specific GRE values can be calculated for every specific type of the REs, when only the TFBS related to the respective RE type are taken into account, e.g., GRE_LR/ERV_, GRE_LINE_, and GRE_SINE_.

To separately assess RE-linked TFBS for each of 225 different TF, we created a table for all human genes and all 225 TFs studied here (Table S2 in Supplementary Material). For each gene, *i* and TF *j* an entry with indices (*i, j*) is number of RE-linked TFBS of this TF in the neighborhood of this gene.

For every individual gene *g*, analogous value termed GTE (Gene TFBS Enrichment) was calculated according to the following formula:

$${\text{GTE}}_{g}=\frac{{\text{TTS}}_{g}}{\frac{1}{n}\sum_{i=1}^{n}{\text{TTS}}_{i}},$$

where TTS*_g_* is total number of TFBS reads mapped in the 5-kb neighborhood of a gene *g*; *n* is the number of all genes; *i* is gene index and TTS*_i_* is the sum of TFBS reads mapped in the 5-kb neighborhood of a gene *i*.

Alternatively, to assess the relative enrichment in RE-linked TFBS for a certain gene compared with the total number of TFBS for the same gene, the normalized value termed NGRE was introduced:

$${\text{NGRE}}_{g}={\text{GRE}}_{g}/{\text{GTE}}_{g}.$$

### Measuring Pathway Enrichment by the RE–Linked TFBS

The gene structures of the human molecular pathways were extracted from the following databases: BioCarta,^10^ KEGG,^11^ NCI,^12^ Reactome,^13^ and Pathway Central.^14^ For each pathway, the PII was calculated according to the formula:

$${\text{PII}}_{p}=\frac{\sum_{i=1}^{n}{\text{GRE}}_{i}}{n},$$

where PII*_p_* is the PII score for a pathway *p*; GRE*_i_* is the GRE score for a gene *i*; and *n* is the number of genes in a pathway *p*. PII*_p_* value is normalized on the number of genes in a pathway to avoid artificially higher values for larger pathways.

PGI (Pathway Gene-based TFBS involvement Index) is expressed by the formula:

$${\text{PGI}}_{p}=\frac{\sum_{i=1}^{n}{\text{GTE}}_{i}}{n},$$

where PGI*_p_* is the PGI score for a pathway *p*, GTE*_i_* is the GTE score for a gene *i*, and *n* is the number of genes in a pathway *p*.

The normalized enrichment in RE-linked TFBS for regulation of a certain molecular pathway termed NPII was calculated as follows:

$${\text{NPII}}_{g}={\text{PII}}_{g}/{\text{PGI}}_{g}.$$

### GO Enrichment Analysis

Gene ontology analysis of the top and the bottom 6% of the genes by GRE scores profiled for all REs was performed using DAVID software.^15^ The *p*-values specifying the significance of observed GO terms and Annotation Clusters enrichment were calculated using a modified Fisher’s exact test (38). The cutoff for *p*-values was set to be equal to 0.05. The enrichment values of GO terms and Annotation Clusters were calculated as fold changes of their occurrence in the sample versus their occurrence in the human genome (38).

### Testing the Significance of the Observed Correlations

The statistical significance of correlations was computed as Pearson correlation coefficient with *p*-value using the Seaborn package.^16^

## Discussion

**Figure 5:**
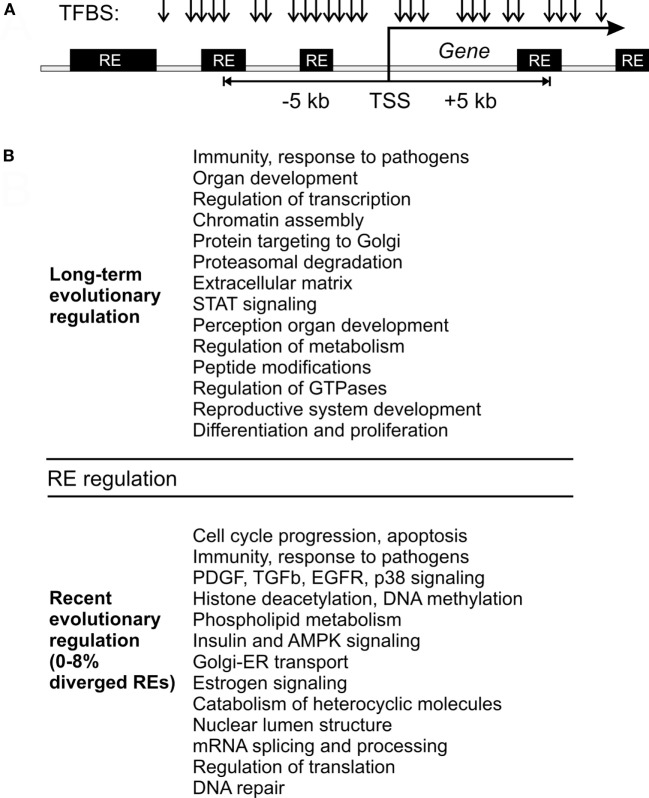
Model of gene expression regulation by RE-linked transcription factor binding site (TFBS). **(A)** Schematic representation of TFBS (arrows) that may overlap with the REs (black boxes) close to transcription start sites of known human genes (shown as “TSS”). **(B)** Outline of RE-linked TFBS regulation at the long-term and recent evolutional perspectives. This figure aggregates data for both single gene and molecular pathway analysis.

Our data strongly evidence that the evolutionary changes in transcriptional regulation of gene expression by REs are tightly associated with the gene functions. From the ENCODE database, we extracted TFBS information for the human leukemia cell line K562. For our analysis, we took the TFBS located in the 5-kb neighborhood of the transcription start sites of known protein-coding genes (Figure 5). Approximately 13 millions TFBS reads were identified meeting these criteria. Among them, ~17% overlapped with the RE sequences and were referred as the RE-specific fraction of TFBS. They were formed by the three major RE classes: ~44% of them were attributed to SINEs; ~33%—to LINEs, and 23%—to LR/ERVs. Some REs are known to be transpositionally competent in the human genome and theoretically could generate a cell line-specific population of the RE inserts. However, they only form a negligible proportion of the RE content and could only exert a minor influence on an overall figure of RE-linked TFBS.

Most of the REs hosting TFBS were highly diverged repeats, and for the evolutionary younger elements (0–8% diverged from their consensus sequence), we identified only ~7% of all RE-specific TFBS. Among them, SINEs covered ~68%, LINEs ~15%, and LR/ERVs ~17% of TFBS (Figure 1). This suggests that in the recent evolutionary horizon SINEs were approximately four times more active than LINEs and LR/ERVs in providing functional TFBS. For the same gene neighborhood, the *young* REs provided functional TFBS generally ~14 times less frequently than the group of *all* REs. These data are congruent with the previously published hypothesis that upon insertion into the host DNA, the newly integrated REs are heavily suppressed. This block is held until they accumulate sufficient number of mutations (48). We show here that the extent of this suppression is different for different RE types varying from ~9-fold for SINEs till ~32-fold for LINEs, with the median level for LR/ERVs. The absolute concentrations for the REs were also different, varying from ~0.1 for LR/ERVs and LINEs till 0.4 for SINEs.

Moreover, LINEs-linked TFBS are more numerous than the SINEs-linked ones outside the gene neighborhoods, whereas the reverse situation ids observed near the genes (Figure 1). Taken together, these data are also supportive toward another hypothesis that the recent genomic inserts of LINEs and LR/ERVs are significantly more deleterious for the human genome than for the SINEs (49, 50).

We calculated the absolute RE-linked TESs for the individual genes and for the molecular pathways. The most strongly affected genes and pathways were implicated in the major processes such as cell stress and immune response, ribosome biogenesis and translation, chromatin remodeling and DNA replication, and organization of mitotic spindle and cell cycle progression. On the other hand, the most weakly regulated genes and pathways were mostly dealing with the embryonic development and neurogenesis (Tables S3 and S4 in Supplementary Material). We next showed that the distribution of RE-linked TFBS generally followed the same trend as the total distribution of all TFBS (Figure 3, Pearson correlation coefficient = 0.47, *p*-value < 0.001). The respective lists of top and bottom implicated processes were also highly interconnected for RE-linked and all TFBS, featuring most strongly regulated protein translation, chromatin remodeling and DNA replication versus most weakly regulated embryonic development and neurogenesis (Table S3 in Supplementary Material). It should be noted that TFBS abundance most likely depends on the importance of a given gene/pathway for the cell type under investigation. For example, for the intensively proliferating leukemia K562 cells investigated here, the programs of embryonic development and neurogenesis can be of an especially low priority, in contrast to DNA replication, protein translation and cell cycle progression (top processes). However, the correlations between all TFBS and RE-linked TFBS features were statistically significant yet not very high (Figure 3). This means that there are many fields where the RE-mediated TFBS regulation is different from the general TFBS distribution rule.

The processes specifically enriched in RE-linked TFBS regulation may be thought the most quickly evolving because RE-linked TFBS are generally not conservative among the different species, unlike those located on the unique segments of DNA (51–53). We next attempted to identify the RE-specific trends in the regulation of gene expression and pathway activation. To this end, we analyzed the relative values of RE-specific TFBS profiles normalized on all TFBS profiles for the same genes (Table S7 in Supplementary Material). Of note, the most strongly specifically RE-regulated protein-coding genes were three different genes for the ubiquitin-specific peptidases, which underline relatively faster evolution of the enclosing molecular processes. The top RE-regulated features were strongly connected with the immunity and response to pathogens, and also with the negative regulation of gene transcription, protein targeting to Golgi, ubiquitination and protein degradation, extracellular matrix organization, regulation of STAT signaling, development and functioning of perception organs and reproductive system, fatty acids metabolism, regulation of GTPase activity, negative regulation of cell differentiation and positive regulation of cell division, and with regulation of body fluids (Tables 3 and 4).

By contrast, the processes most weakly regulated by the REs were linked mostly with the embryonic development, stem cell differentiation, nerve growth and neuronal signaling, cytokine signaling networks, transcription and processing of RNA, nuclear chromatin organization, ribosome assembly and protein translation, IGF1R signaling, and regulation of glucose metabolism (Tables 3 and 4).

Moreover, the RE-specific TESs can be calculated for the different fractions of the REs. Here, we analyzed their distributions for the evolutionary *young* fraction of the REs (diverged less than 8%), and for *all* REs. The regulation features in the *all* RE fraction demonstrate long-term tendencies in RE-specific accumulation of TFBS, whereas the *young* fraction may serve as the marker for the relatively recent trends in the human genome evolution, starting roughly since the radiation of Old World monkeys (7, 54). Both gene- and pathway-specific scores statistically significantly correlated for the *young* and *all* RE-linked TFBS (Figure 4). This suggests that the major evolutional trends in RE-linked TFBS regulation are largely conserved. Interestingly, the pathway-specific score was correlated stronger than the gene-specific score (Figure 4A, Pearson correlation coefficient = 0.38, *p*-value < 0.001; Figure 4B, Pearson correlation coefficient = 0.57, *p*-value < 0.001), which is in line with the previous findings that the data aggregation at the level of molecular pathways provides more stable results and may enhance correlations compared with the single-gene level of analysis (41).

To analyze differences in gene and pathway regulation by *all* and *young* REs, we calculated ratios of the above gene- and pathway-specific scores for *all* and *young* REs. Bigger values here mean greater regulation changes in a long-term rather than recent evolution, lower values—by contrast, greater changes in the recent rather than long-term evolution (Table 4; Tables S10 and S11 in Supplementary Material). In the long-term, but not short-term perspective, the top evolving pathways were linked mainly with the immunity and cytokine signaling, cell adhesion, Notch, Wnt, and integrin signaling, neuronal development and sensing, chondroitin sulfate and heparin metabolism, cAMP metabolism, endocytosis, and IGF1R signaling.

By contrast, the most quickly recently evolving processes were linked mainly with the immunity, cell cycle progression and apoptosis attenuation, PDGF, TGF beta, EGFR, and p38 signaling, histone deacetylation and DNA methylation interplay, structure of nuclear lumen, metabolism (primarily catabolism) of phospholipids and heterocyclic nitrogen-containing molecules, insulin and AMPK signaling, retrograde Golgi-ER transport, estrogen signaling, and oocyte maturation (Figure 5). The immunity-linked pathways were highly represented in both categories (recently and long-term evolving), but their functional characteristics were different and did not overlap (Table 5). These pathways are mostly connected with inflammation, pathogen recognition of innate immunity and cytokine signaling. Our findings concerning the RE impact of the both long-term and short-term evolution of human immune system are in accord with recent experimental findings that HERV have dispersed numerous IFN-inducible enhancers regulating essential innate immune functions (10, 11).

**Table 5:** Top and bottom immunity-linked molecular pathways sorted by the ratios of NPII scores for the *all* and *young* RE-linked transcription factor binding site.

Pathway name	All NPII	Young NPII	atio (A/Y)
**Top ratio pathways**			

NCI downstream signaling in naive CD8 T cells pathway (pathway regulation of survival gene product expression *via* IL2RG)	1.02	0.18	5.59
NCI downstream signaling in naive CD8 T cells main pathway	0.93	0.43	2.16
Reactome toll like receptor 4 TLR4 cascade main pathway	1.19	0.32	3.72
Reactome interleukin receptor SHC signaling main pathway	0.85	0.24	3.51
Cytokine network pathway	1.04	0.32	3.24
NCI CXCR3 mediated signaling events pathway (cell adhesion)	1.06	0.39	2.76
NCI CXCR3 mediated signaling events pathway (actin polymerization or depolymerization)	1.02	0.40	2.57
NCI LPA receptor mediated events pathway (cAMP biosynthetic process)	0.87	0.35	2.46
Biocarta lck and fyn tyrosine kinases in initiation of tcr activation main pathway	0.98	0.42	2.36
NCI IL2 mediated signaling events pathway (T cell proliferation)	1.00	0.46	2.19
NCI BCR signaling pathway (reentry into mitotic cell cycle)	0.88	0.42	2.12
NCI IL4-mediated signaling events main pathway	0.96	0.46	2.08
KEGG inflammatory bowel disease IBD main pathway	1.01	0.49	2.06

**Lower ratio pathways**			

Reactome IRAK2 mediated activation of TAK1 complex main pathway	1.17	1.40	0.84
Reactome IRAK2 mediated activation of TAK1 complex upon TLR7 8 or 9 stimulation main pathway	1.17	1.40	0.84
KEGG Fanconi anemia main pathway	0.98	1.17	0.83
Reactome Fanconi anemia main pathway	0.89	1.08	0.82
Reactome CD28 dependent Vav1 main pathway	1.13	1.37	0.83
Reactome thromboxane signaling through TP receptor main pathway	0.94	1.15	0.82
NCI Thromboxane A2 receptor signaling pathway (JNK cascade)	0.95	1.20	0.79
NCI Fc epsilon receptor I signaling in mast cells pathway (regulation of mast cell degranulation)	0.82	1.03	0.79
IL-10 pathway IL-10 responsive genes transcription of BCLXL cyclin-D1 D2 D3 Pim1 c-myc and P19 (INK4D) *via* STAT3	0.99	1.31	0.76
IL-10 pathway inflammatory cytokine genes expression *via* STAT3	0.99	1.31	0.76
Reactome membrane binding and targeting of GAG proteins main pathway	0.94	1.25	0.75

Patterns of genes mostly impacted by transposons are generally consistent with universals of genome evolution (55). Our findings of RE-impacted changes in human molecular pathways are also generally in line with both ancient and recent trends in the evolution of human lineage. Retrotransposon insertion is an abrupt event that can drastically affect expression of neighboring genes by regulatory innovation and direct mutation (9). A general hypothesis was proposed that genes that are highly expressed in all tissues (mostly cytoplasmic and housekeeping) cannot tolerate regulatory and mutational pressure imposed by transposons without fitness loss (56, 57) because the toxic effects of protein misfolding and stoichiometric imbalance of subunits are thought to be most severe for highly abundant proteins (58). Here, we show that human RE impact mainly the pathways linked with immunity, signal transduction, proliferation, cell interaction and communication both on the recent and the long-term time scales, whereas cytoplasmic and housekeeping molecular pathways are weaker affected.

Moreover, evolutionary history of human lineage most likely includes series of time-periods with the accelerated evolution of some particular molecular systems, i.e., due to evolutionary arms race (59), run-away processes of sexual selection (60), and classical positive selection, e.g., selection for the ability to accept new types of food (61). Interestingly, regulatory innovations were probably the major source of changes throughout the recent human evolution (62). First, evolutionary arms-race between human ancestors and various pathogens has driven the changes of adaptive immune response (63) and is still shaping human immunity nowadays (64). Here, we show that such shaping is mediated also through RE insertions and exaptation of their TFBS to regulate expression of immunity-linked genes. Interestingly, long-term and short-term evolutionary pressures onto the human immune system sometimes appear disjoined, e.g., because of encountering new pathogens, reflected by the fact that different modules of immunity were affected by REs on different time scales (Table 5). Second, evolution of human brain was largely affected by sexual selection under a trend toward monogamy, lowering male competition, and increasing female choice (65). Our study suggests that REs had been affecting human nerve system for a long time (Table 4) that may accounts for multiple events in the evolution of mammalian brain. Third, recent human evolution after divergence with chimpanzee imposed several dietary transitions, such as increased meat-eating that occurred ~2 mya simultaneously with massive usage of stone and fire (61). Therefore, recent changes in the catabolism of heterocyclic molecules and phospholipid catabolism can be at least partly connected with this kind of food speciation of great apes and hominids. Fourth, rapid recent RE-affected evolution of histone deacetylation and DNA methylation interplay can be at least partly connected with gradual diversification of transposon-repressing KRAB zinc finger TFs (66), reflecting intragenome evolutionary arms race between REs and host genes.

In this study, we analyzed in depth RE-linked TFBS signatures for a unique human cell line where the high-throughput TFBS profile is currently available. Further accumulation of high-throughput data on TFBS distribution will make it possible to build a more robust model of RE influence on human molecular pathways based on thorough analysis of many objects including various cell lines and hopefully intact and pathological human tissues.

Finally, given that REs make up >40% of genomic sequence and that >80% of the REs are located outside promoter-neighboring regions, it remains of a great interest to further investigate if this larger subset of REs may have significant role in the evolution of human molecular pathways that can be mediated *via* chromatin remodeling or regulation of non-coding RNAs. This will be a matter of further investigation in our consortium.

## Author Contributions

DN, DP, and AG analyzed transcription factor binding sites (TFBS) data; MS constructed molecular pathways library; VT mapped TFBS and retrotransposons on human genome; NB, AP, VP, and AB wrote and implemented algorithms for data analysis; and AB and DN wrote the paper.

## Conflict of Interest Statement

The authors declare that the research was conducted in the absence of any commercial or financial relationships that could be construed as a potential conflict of interest.

## Footnotes

^1^www.geneontology.org.

^2^https://www.encodeproject.org/chip-seq/transcription_factor/.

^3^https://www.encodeproject.org/pipelines/ENCPL220NBH/.

^4^https://www.encodeproject.org.

^5^http://www.repeatmasker.org.

^6^https://genome.ucsc.edu/cgi-bin/hgTables.

^7^https://genome.ucsc.edu/goldenpath/help/bedgraph.html.

^8^https://www.encodeproject.org/pipelines/ENCPL138KID/.

^9^https://genome.ucsc.edu/cgi-bin/hgTables.

^10^https://cgap.nci.nih.gov/Pathways/BioCarta_Pathways.

^11^http://www.genome.jp/kegg/.

^12^https://cactus.nci.nih.gov/ncicadd/about.html.

^13^http://reactome.org.

^14^http://www.sabiosciences.com/pathwaycentral.php.

^15^https://david.ncifcrf.gov/.

^16^http://seaborn.pydata.org/.

## Supplementary Material

Table S1 GRE score for REs of different classes and evolutionary ages.

Table S2 Number of RE-linked transcription factor binding sites for human genes and 225 analysed transcription factors.

Table S3 GO terms for top and bottom 6% of genes by GRE of all REs.

Table S4 PII score for all REs for human molecular pathways.

Table S5 PII score for all REs for top and bottom 65 human molecular pathways.

Table S6 GTE score for human genes.

Table S7 NGRE score for all and young REs.

Table S8 GO terms for top and bottom 6% of genes by NGRE of all REs.

Table S9 Comparison of NPII for all and young REs.

Table S10 Ratio of all NGRE and young NGRE for human genes.

Table S11 Ratio of all NPII and young NPII for human molecular pathways.

Table S12 List of studied transcription factors.

Table S13 List of data files corresponding to studied transcription factors.
